# Unbiased biocatalytic solar-to-chemical conversion by FeOOH/BiVO_4_/perovskite tandem structure

**DOI:** 10.1038/s41467-018-06687-z

**Published:** 2018-10-11

**Authors:** Yang Woo Lee, Passarut Boonmongkolras, Eun Jin Son, Jinhyun Kim, Sahng Ha Lee, Su Keun Kuk, Jong Wan Ko, Byungha Shin, Chan Beum Park

**Affiliations:** Department of Materials Science and Engineering, Korea Advanced Institute of Science and Technology (KAIST), 335 Science Road, Daejeon, 305-701 Republic of Korea

## Abstract

Redox enzymes catalyze fascinating chemical reactions with excellent regio- and stereo-specificity. Nicotinamide adenine dinucleotide cofactor is essential in numerous redox biocatalytic reactions and needs to be regenerated because it is consumed as an equivalent during the enzymatic turnover. Here we report on unbiased photoelectrochemical tandem assembly of a photoanode (FeOOH/BiVO_4_) and a perovskite photovoltaic to provide sufficient potential for cofactor-dependent biocatalytic reactions. We obtain a high faradaic efficiency of 96.2% and an initial conversion rate of 2.4 mM h^−1^ without an external applied bias for the photoelectrochemical enzymatic conversion of α-ketoglutarate to l-glutamate via l-glutamate dehydrogenase. In addition, we achieve a total turnover number and a turnover frequency of the enzyme of 108,800 and 6200 h^−1^, respectively, demonstrating that the tandem configuration facilitates redox biocatalysis using light as the only energy source.

## Abstract (web summary)

Photoelectrochemical (PEC) cell platforms typically need an electrical bias that drives the electron transfer from the photoanode to the photocathode. Here, the authors report a bias-free PEC tandem device for solar-driven redox biocatalysis.

## Introduction

In nature, green plants harvest solar energy through the Z-scheme for the biocatalytic synthesis of high-energy chemicals during the Calvin cycle. From an industrial point of view, redox enzymes are useful catalysts that can accelerate many complex reactions with excellent specificity under mild conditions^1,2^. Despite such competence, wider applications of many oxidoreductases are often limited due to the need for an expensive nicotinamide cofactor, NAD(P)H^3^. The solar regeneration of NAD(P)H cofactors from its oxidized form [i.e., NAD(P)^+^] via photoelectrochemical (PEC) means can sustainably provide reducing power for activating redox biocatalysts in a similar way to natural photosynthesis^4^. The PEC platform is superior to photochemical ones due to its directional electron transfer, its better operating stability, and the recyclability of photoelectrodes for repeated reactions^5^. However, generating a bias large enough to drive the desired PEC reaction from a single light-absorbing layer remains challenging^6^. Previously, we built biocatalytic PEC platforms in photoanode/photocathode tandem configurations, such as triple-junction silicon/hydrogen-terminated silicon nanowire^7^ and FeOOH-Fe_2_O_3_/BiFeO_3_^8^, but an additional bias as high as 1.2 V was always required to promote NAD(P)H-dependent biocatalytic reactions^9^. Instead of applying an external bias, the integration of a photovoltaic device in a series with a photoelectrode can solve the issue by capturing the unabsorbed light at the photoanode^10^. For example, Krol et al. recently reported the combination of W-doped BiVO_4_ photoanode and a 2-junction a-Si solar cell for unbiased PEC water splitting^11^.

**Fig. 1:**
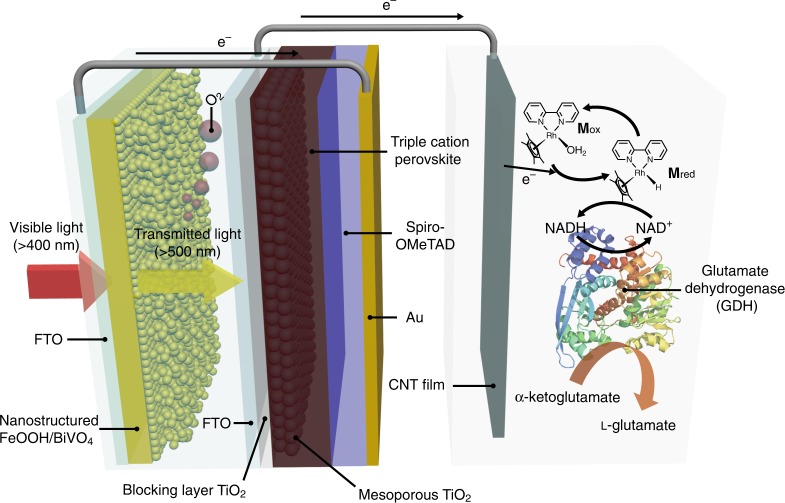
Graphical illustration of unbiased PEC biocatalysis using a tandem configuration. The FeOOH/BiVO_4_/perovskite tandem structure promotes PEC water oxidation and the CNT film cathode provides photoexcited electrons for the regeneration of NADH cofactors to be coupled with redox enzymatic reaction by GDH

Here, we report unbiased solar NAD(P)H regeneration and redox biocatalysis using a large-scale, tandem PEC configuration consisting of a nanostructured FeOOH/BiVO_4_ photoanode, an organometallic perovskite-based photovoltaic cell, and a carbon nanotube (CNT) film cathode. As depicted in Fig. 1, FeOOH is applied as a water oxidation catalyst to the BiVO_4_ photoanode to enhance the extraction of photogenerated holes and the efficiency of water oxidation, as well as to improve the photoanode’s stability. The perovskite solar cell with a light absorber containing triple cations, Cs, formamidinium, and methylammonium absorbs the transmitted light through the FeOOH/BiVO_4_ photoanode, providing additional photovoltage to satisfy the thermodynamic requirement for both water oxidation and the regeneration of NADH cofactors. For the efficient regeneration of NADH from NAD^+^, we adopt conductive CNT film as a cathode for the reduction of an Rh-based electron mediator **M** {[Cp*Rh(bpy)H_2_O]^2+^, Cp* = C_5_Me_5_, bpy = 2,2′-bipyridine}, which reduces NAD^+^ to enzymatically active 1,4-NADH cofactor and prevents the formation of inactive 1,6-NADH and NAD_2_ dimer. We successfully regenerate NADH cofactors in an enzymatically active form, which then take part in the conversion of α-ketoglutarate to l-glutamate via glutamate dehydrogenase (GDH), an NADH-dependent redox enzyme.

## Results and Discussion

### Characterization of FeOOH/BiVO_4_ photoanode

**Fig. 2:**
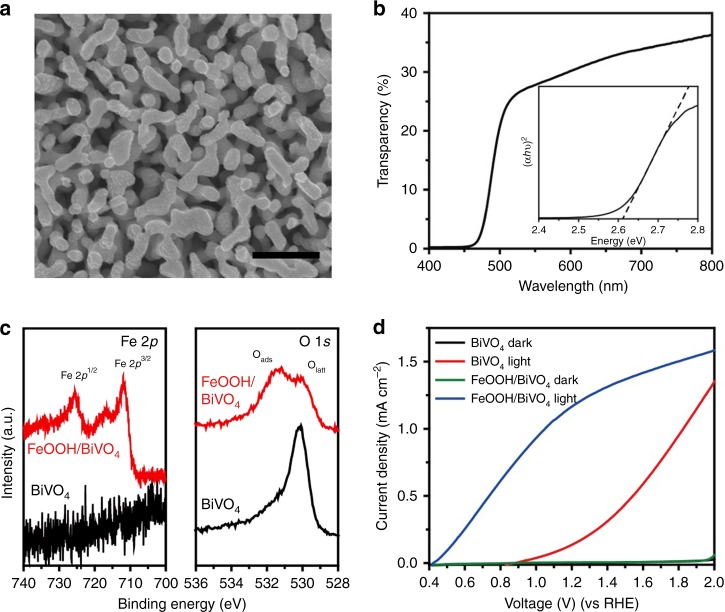
Characterization of FeOOH/BiVO_4_ photoanode. **a** SEM image of FeOOH/BiVO_4_ (top view, scale bar: 500 nm). **b** Transparency of FeOOH/BiVO_4_ measured by a UV-Vis spectrometer and a Tauc plot (inset) of BiVO_4_ indicating 2.6 eV of bandgap. **c** XPS local scan spectra of Fe 2*p*, O 1*s* regions before (black) and after (red) the deposition of FeOOH on BiVO_4_. **d** PEC *I*–*V* characteristics of the BiVO_4_ photoanode with (blue) and without (red) a FeOOH catalyst. The active area of the photoanode was 1 cm^2^

We prepared a nanostructured FeOOH/BiVO_4_ photoanode according to the literature^12^. The plan-view scanning electron microscopic image of FeOOH/BiVO_4_ in Fig. 2a clearly illustrates the formation of a nanostructure consisting of agglomerated particles 120 nm in size. The optical bandgap of nanostructured BiVO_4_ determined based on a Tauc plot in Fig. 2b was approximately 2.6 eV, which is consistent with literature values^13^. The formation of the FeOOH was confirmed via X-ray photoelectron spectroscopic analysis (Fig. 2c), which revealed the appearance of Fe 2*p* peaks and an O 1*s* peak at 531.4 eV^14^ after the electrodeposition of FeOOH. Note that a negligible change in the transparency of the BiVO_4_ electrode occurred after the electrodeposition of the FeOOH catalyst (Supplementary Figure 1). We carried out a half-cell PEC measurement to evaluate photocatalytic performance of the BiVO_4_ photoanodes (Fig. 2d). We should emphasize that the active area of the BiVO_4_ photoanode used for this study including Fig. 2d was 1 cm^2^, which is larger than the typical areas used in previous studies on PEC water splitting with a BiVO_4_ photoanode^15,16^. As depicted in Supplementary Figure 2, a comparison of two PEC half-cells with the BiVO_4_ photoanodes of different active areas (1 vs 0.2 cm^2^), which were prepared in the same batch, clearly demonstrate the improved PEC performance with the smaller device size. However, our study’s aim was to demonstrate a route to a large-scale unbiased PEC device for redox biocatalysis, and, therefore, the larger-area BiVO_4_ photoanode was used for the tandem structure. The high onset potential (0.9 V vs RHE) of the bare BiVO_4_ was shifted in the cathodic direction to 0.4 V, which confirms the successful deposition of the FeOOH catalyst and its catalytic activity in reducing the overpotential of the water oxidation reaction. Concomitantly, the current density at 1.23 V significantly increased from 0.15 to 1.19 mA cm^−2^.

### Photovoltaic performance of perovskite solar cell

**Fig. 3:**
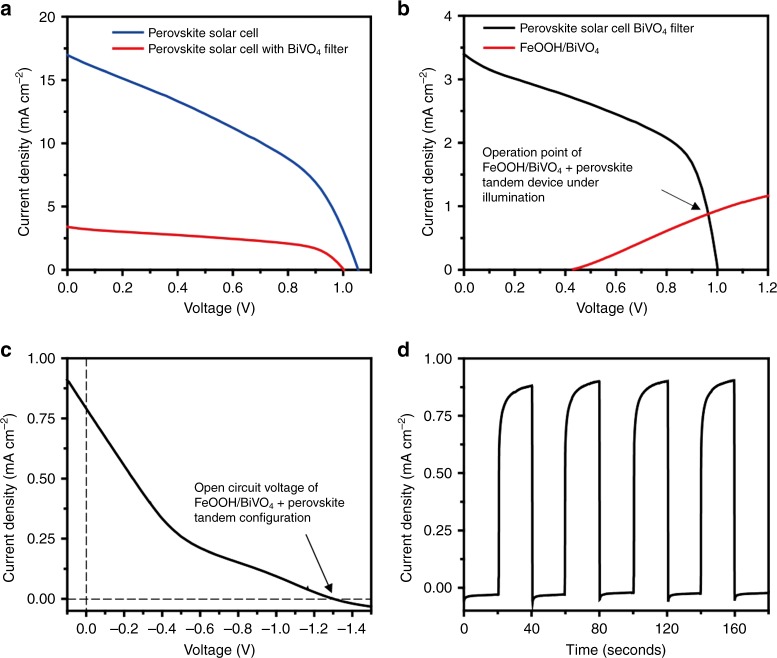
Performances of the perovskite solar cell and the PEC tandem configuration. **a** *I*–*V* characteristics of perovskite solar cell under illumination (AM 1.5 G, 100 mW cm^−2^) with (red) and without (blue) BiVO_4_ filter. The active area of solar cell was 1 cm^2^. **b** Overlap of *I*–*V* profiles of FeOOH/BiVO_4_ photoanode (red) and perovskite solar cell under filtered illumination (black). The intersection of two graphs indicates an operation power of the tandem device. **c** Linear sweep voltammetry of the tandem device in a two-electrode configuration (i.e., FeOOH/BIVO_4_/perovskite and Pt wire). The voltage at zero current density designates the open-circuit voltage of the tandem system under illumination. **d** Photocurrent density profile of FeOOH/BiVO_4_/perovskite solar cell with a two-electrode configuration

To eliminate the need for external bias to drive the target PEC reaction, we used a triple-cation organometallic perovskite solar cell^17^ as an additional power source to couple with the FeOOH/BiVO_4_ photoanode. The optical bandgap of the triple-cation perovskite was estimated to be 1.5 eV, which is within the ideal range for forming a tandem structure with the light absorber of a 2.6 eV bandgap (i.e., BiVO_4_). The current density–voltage (*I*–*V*) characteristic of a small-area (0.1 cm^2^) device is illustrated in Supplementary Figure 3. The device exhibits a photoconversion efficiency of 16.1%, a short circuit current of 21.4 mA cm^−2^, and an open-circuit voltage of 1.07 V with slight hysteresis, which is commonly observed in perovskite solar cells^18^. Note that the data in Supplementary Figure 3 were measured using a perovskite solar cell with an active area of 0.1 cm^2^, similarly to many other studies that reported high-performance perovskite solar cells^19^. To match the size of the FeOOH/BiVO_4_ photoanode, we further proceeded to fabricate a larger-sized perovskite solar cell (active area: 1 cm^2^). As expected, a significant decrease of the photovoltaic performance occurred upon the scaling up of the active area; the photoconversion efficiency dropped to 7.19%. A major contributing factor in the deterioration of the efficiency was the poor fill factor (71.7% for the 0.1 cm^2^ device vs 46.7% for the 1 cm^2^ device). This stemmed from a higher series resistance of the larger device originating from a mediocre level of the lateral electrical conductivity of the transparent FTO electrode compared with a metal electrode; electrons photogenerated far from the contacted area of the FTO to the external circuit will face difficulty when it comes to getting extracted. When an *I*–*V* curve was measured with the light source filtered via the FeOOH/BiVO_4_ photoelectrode, a condition that is more relevant to the analysis of a tandem device, the efficiency dropped to 2.23% with a lower current density of 3.4 mA cm^−2^ as indicated by the red curve in Fig. 3a. The fill factor of the same perovskite solar cell improved to 66.8% from 46.7% with the light filtering. This is because the importance of the series resistance became less relevant when a smaller amount of photocurrent was flowing. The large open-circuit voltage of 0.98 V was maintained, the value of which was large enough to drive the PEC reaction when coupled with the FeOOH/BiVO_4_ photoanode.

### Photoelectrochemical behavior of the tandem structure

To validate the light-induced current density and the voltage generation by FeOOH/BiVO_4_ and perovskite solar cell in the tandem configuration, we first conducted PEC water splitting reaction using a Pt wire as a cathode for hydrogen evolution. To estimate the operating current of the tandem device, we constructed a plot overlaying the *I*–*V* curves of the FeOOH/BiVO_4_ photoanode and the perovskite solar cell with the filtered illumination (Fig. 3b). The operating current density of the tandem configuration was estimated to be 0.87 mA cm^−2^ from the intersection of the two curves. This is consistent with the actual photocurrent measured from the tandem device, 0.82 mA cm^−2^ (Fig. 3c, d). As displayed in Fig. 3c, our linear sweep voltammetric measurement under the one sun illumination of a two-electrode configuration along the cathodic direction reveals that the open-circuit voltage (1.3 V) is larger than the potential required to promote both water oxidation at the photoanode and NADH regeneration at the cathode. Because the reduction potential of the electron mediator, **M** (−0.73 V vs Ag/AgCl)^20^, is near the reduction potential of molecular hydrogen, the tandem configuration can readily drive the reduction of **M**.

### Characterization of CNT film cathode

**Fig. 4:**
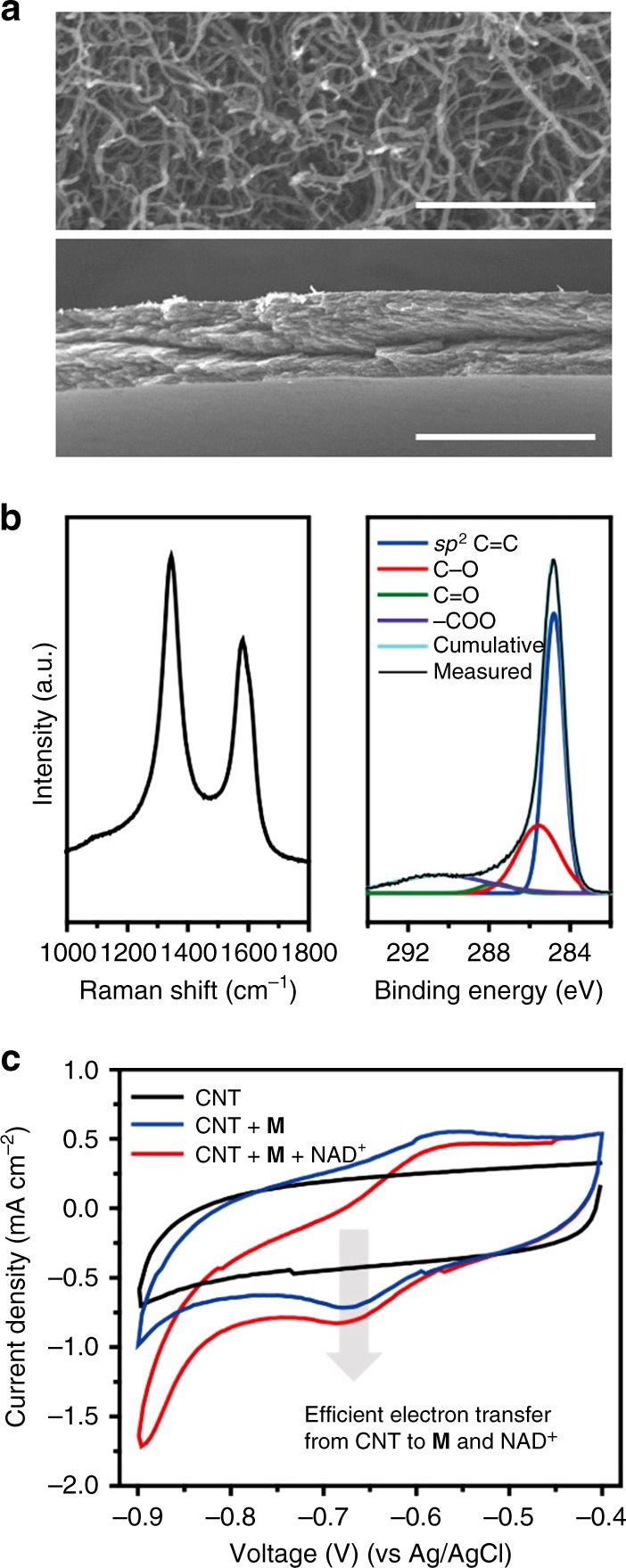
Characterization of CNT film cathode. **a** SEM images of CNT film in top view (upper image, sclae bar: 500 nm) and cross-section (lower image, scale bar: 100 μm). **b** Raman spectrum (left) and XPS local scan spectrum of C1*s* region (right) of CNT film. **c** The changes of the cyclic voltammogram of CNT film (black) by the addition of **M** (blue) and NAD^+^ (red). Experimental conditions: 0.5 mM **M**, 1 mM NAD^+^ in a phosphate buffer (0.1 M, pH 7.5), scan rate: 5 mV s^−1^

As a cathode material for the reduction of NAD^+^, we fabricated CNT films via a vacuum-filtrating method, which is simple and cost effective. The CNT cathode was connected to a copper wire with silver paste to make ohmic contact with the bottom electrode of the perovskite device; an active area of 1 cm^2^ was left uncovered from the epoxy resin. Our SEM analysis revealed that CNTs formed a highly porous network with a film thickness of ~50 μm (Fig. 4a). The intensity ratio of the D-band to G-band in the Raman spectrum was approximately 1.36, indicating the multi-walled nature of our CNT electrode. The C 1*s* XPS peak from the CNT film indicated the presence of hydroxyl (C–OH), carbonyl (C=O), and carboxyl (COO) groups (Fig. 4b), which are typical functionalities of multi-walled CNTs^21^. These results confirm no significant alteration in the properties of CNTs during the filtrating process. We observed the reduction of **M** via the CNT cathode using linear sweep voltammetry in a typical three-electrode configuration: CNT cathode as a working electrode, Ag/AgCl as a reference electrode, and Pt wire as a counter electrode. The *I*–*V* curves from the CNT film in the solution containing **M** revealed an increase of the cathodic current near the applied bias of −0.75 V (vs Ag/AgCl, pH 7.5) (Fig. 4c), which indicates the reduction of **M** at the surface of the CNT film. The measured reduction potential of **M** at −0.75 V is slightly higher in magnitude compared with the thermodynamic hydrogen evolving potential at pH 7.5 (i.e., −0.64 V vs Ag/AgCl). In addition, the cathodic current near −0.75 V further increased with the addition of NAD^+^, which is attributed to the sequential transfer of electrons from CNT to **M** and further to NAD^+^. As shown in Supplementary Figure 4, however, direct reduction of NAD^+^ (without **M**) by the CNT cathode was also observed at −1.2 V (vs Ag/AgCl), which should cause the formation of enzymatically inactive NAD_2_ dimer or NADH isomers (i.e., 1,2-NADH, 1,6-NADH) through the side reactions depicted in Supplementary Figure 5A. To avoid the direct reduction of NAD^+^ by the CNT cathode, the mediation by **M** is essential to transfer a hydride to NAD^+^ to form active 1,4-NADH^22^ at lower overpotential of −0.76 V (vs Ag/AgCl)^23^ as illustrated in Supplementary Figure 5B.

The electron transfer from the CNT cathode to **M** was further investigated via cyclic voltammetric analysis with varying scan rates (Supplementary Figure 6A). In the cyclic voltammograms, capacitive charging current was observed due to the double-layer formation at the surface of the CNT film in the aqueous electrolyte^24,25^. We deconvoluted the faradaic current by the redox reaction from the measured cyclic voltammogram (Supplementary Figure 6B) to evaluate the electrochemical interaction between the CNT film and **M**. The cathodic peak currents of the CNT electrode was proportional to the scan rate (Supplementary Figure 6C), suggesting that the reaction was not limited by diffusion. This is in agreement with Laviron’s theory, which predicts that direct charge transfer occurs only between the electrode and absorbates^26^; **M** molecules are adsorbed on the surface of CNT^27^ via π–π interaction and cation–π interaction. From the linear dependency of the cathodic peak potentials on the logarithm of the scan rate in the presence of **M** (Supplementary Figure 6D), we estimated the charge transfer coefficient (*α*) and the heterogeneous electron transfer rate constant (*k*_s_) as 0.119 and 0.242, respectively.

### Unbiased photoelectrochemical regeneration of NADH

**Fig. 5:**
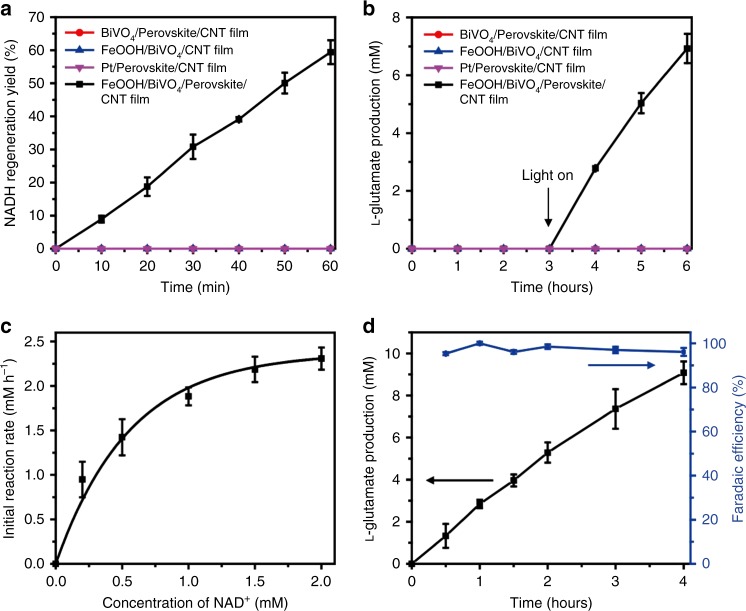
Bias-free photoelectrochemical NADH regeneration and biocatalytic reactions. **a** Regeneration yield of NADH in the absence of the perovskite solar cell and FeOOH/BiVO_4_ photoanode, and in the tandem configuration with both photoanode and photovoltaic module under illumination. **b** Biocatalytic production of l-glutamate in the absence of the perovskite solar cell and FeOOH/BiVO_4_ photoanode, and in the tandem configuration with both photoanode and photovoltaic module under illumination. **c** Influence of NAD^+^ concentration on the initial reaction rate of PEC biocatalytic production of l-glutamate under illumination. **d** Time profile of biocatalytic production of l-glutamate (black) in the tandem configuration with NAD^+^ (2 mM) and the estimated faradaic efficiency (blue) of the PEC biocatalytic production of l-glutamate from the total charge flowed through the tandem system (mean ± standard deviation, *n* = 3)

We assembled a tandem device consisting of the FeOOH/BiVO_4_/perovskite solar cell and the CNT-based cathode. The water oxidation reaction at the FeOOH/BiVO_4_ and the biocatalytic reduction reaction at the CNT film proceeded in two separate compartments connected by a salt bridge. This was done to prevent the undesirable oxidation of reduced **M**, NADH, and l-glutamate, which could have reduced the efficiency of the enzymatic reaction through the photoexcited holes generated at the surface of the FeOOH/BiVO_4_ photoanode. We note that the photocurrent under the two-compartment configuration was reduced to approximately 33% of the value obtained from the one-compartment configuration (Supplementary Figure 7), which is attributed to the ionic resistance of the salt bridge connecting the two separated reaction vessels. At zero applied bias, 58% of NADH was regenerated from NAD^+^ in 1 h, whereas no regeneration was observed in the absence of the FeOOH catalyst, BiVO_4_ photoanode, or perovskite photovoltaic module (Fig. 5a). As displayed in Supplementary Figure 8, the regeneration rate of NADH was proportional to the concentration of NAD^+^, which indicates higher reducing capability of our tandem configuration at higher NAD^+^ concentration. This level of NADH regeneration efficiency is comparable to the previously reported biocatalytic PEC platform of CoPi/a-Fe_2_O_3_|BiFeO_3_ operating under 1.0 V of an applied bias and an additional chemical bias (created by the pH difference between two compartments)^8^.

### Bias-free photoelectrochemical biocatalysis

We further applied the photoelectrochemical NADH regeneration to the GDH-catalyzed conversion of α-ketoglutarate to l-glutamate to confirm that the regenerated NADH cofactor served as a hydride-donating shuttle to redox enzymes. l-Glutamate was synthesized only through the complete tandem configuration (i.e., FeOOH/BiVO_4_/perovskite/CNT film) upon light irradiation (Fig. 5b). In the absence of the electron mediator, **M**, NAD^+^ reduction (27% yield for 1 h) by the PEC tandem operation was much less efficient than that (58% yield for 1 h) with **M** (Supplementary Figure 9A). Furthermore, the initial rate of enzymatic production of l-glutamate was 25 times slower in the absence of **M**, which indicates that the most of the electrochemically reduced NAD^+^ without **M** were enzymatically inactive (Supplementary Figure 9**b**). Supplementary Figure 10 shows an apparent saturation kinetics of l-glutamate synthesis in terms of **M** concentration, indicating that 0.5 mM is an optimum concentration of **M** for the GDH-driven PEC biocatalysis. We investigated the reaction kinetics by varying the concentration of NAD^+^ in the cathodic compartment where the CNT electrode resided. As displayed in Fig. 5c, the initial rate of l-glutamate synthesis vs NAD^+^ concentration followed a typical Michaelis–Menten kinetic profile. The production yield of l-glutamate decreased with the increasing ratio of substrate/cofactor (Supplementary Figure 11A), which we ascribe to the dependency of NADH regeneration rate on NAD^+^ concentration (Supplementary Figure 8). However, the turnover frequency of NAD^+^ increased with the increasing ratio (Supplementary Figure 11B), which implies high-performance per cofactor at the low cofactor concentration. Figure 5d reveals a time profile of l-glutamate production by the tandem PEC configuration with 50 mM α-ketoglutarate and 2 mM NAD^+^, which shows 2.4 mM h^−1^ of initial conversion rate. The result indicates that our tandem system is capable of a highly efficient biocatalytic reaction through the fast NADH regeneration. Furthermore, we obtained a faradaic efficiency of 96.2% according to Eq. (1):

1 $$	\hskip 80pt{\mathrm{Faradaic}}\,{\mathrm{efficiency}} \\ 	= \frac{{{\mathrm{Charges}}\,{\mathrm{consumed}}\,{\mathrm{for}}\,{\mathrm{converting}}\,{\mathrm{substrate}}\,{\mathrm{to}}\,{\mathrm{product}}}}{{{\mathrm{Total}}\,{\mathrm{charges}}\,{\mathrm{that}}\,{\mathrm{passed}}\,{\mathrm{in}}\,{\mathrm{the}}\,{\mathrm{PEC}}\,{\mathrm{cell}}}}.$$

We attribute the small faradaic loss (i.e., 3.8%) to the multiple electron transfer steps from the CNT cathode to **M** and NAD^+^ in the cathodic compartment^8^ and electrode fouling^28^ by GDH, which may block the active sites on the CNT film cathode. Nonetheless, the high faradaic efficiency value proves that most electrons generated from water oxidation at the photoanode were consumed to regenerate enzymatically active NADH and to convert α-ketoglutarate to l-glutamate.

Next, we investigated long-term stability of the tandem PEC biocatalysis. Supplementary Figure 12 shows a maximum production yield of 81.5% for 48 h by the tandem PEC biocatalytic system. The total turnover number (TTN_GDH_) and the turnover frequency (TOF_GDH_) of GDH were estimated to be 1.08 × 10^5^ and 6.2 × 10^3^ h^−1^, respectively. The values were approximately 58 and 14 times higher, respectively, than those of the previous NADH-dependent PEC biocatalytic systems composed of dual photoelectrodes under an external applied bias^29^. As shown in Supplementary Figure 13A, the enzymatic conversion rate gradually decreased for 48 h irrespective of NAD^+^ and substrate concentrations. We attribute the reduction of conversion rate to (1) potential deactivation of the enzyme over time and (2) the progressive decrease of the current density of the tandem PEC system (Supplementary Figure 13B). The current density decrease is mainly caused by the photodegradation of the perovskite solar cell, a known issue in the photovoltaic community^30^. Further improvement of the tandem PEC system would be achieved by improving the stability of a perovskite solar cell against the environment^31^.

We further compared the tandem PEC biocatalysis at high substrate concentrations with the enzymatic NADH recycling method that used a formate dehydrogenase (FDH) from *Candida boidinii* as a secondary enzyme^32–34^. With the increasing substrate concentration, the PEC-based l-glutamate production yield (Supplementary Figure 14A) and the initial reaction rate (Supplementary Figure 14B) increased but became steady at over 50 mM of α-ketoglutarate concentration. The tandem PEC system was comparable to the enzymatic NADH recycling system driven by 0.2 U ml^−1^ FDH in terms of NADH regeneration rate (Supplementary Figure 15A) and l-glutamate production rate (Supplementary Figure 15B). The leveling-off behavior at high substrate concentration and relatively low performance compared to the enzymatic cofactor recycling system are attributed to the limited number of electrons generated by the PEC tandem system under illumination. The limitations would be solved by enhancing the current generation through the design of more efficient photoelectrodes; for example, by (i) adopting more efficient cocatalysts that have been demonstrated to work well for metal-doped BiVO_4_ photoanodes to enhance water oxidation kinetics^35,36^; (ii) optimizing the morphology of BiVO_4_ to increase the transmittance of longer wavelength by suppressing light scattering, which would allow more light to reach the perovskite solar cell underneath^37,38^; and (iii) improving the stability and performance of perovskite solar cells^39^. Overall, the photoelectrochemical approach is in its infancy but is a promising strategy that utilizes abundant and clean resources of solar energy and water.

In summary, we have demonstrated unbiased PEC biocatalysis by assembling a single-pass tandem configuration that consists of a FeOOH/BiVO_4_ photoanode, organometallic perovskite solar cell, and CNT film cathode for the NADH cofactor regeneration and enzymatic conversion of α-ketoglutarate to l-glutamate via GDH. The active area of the photoanode was the largest among ever reported for solar water splitting. Without the assistance of an external applied bias, the FeOOH/BiVO_4_ photoanode and perovskite solar cell were capable of extracting electrons from water molecules whose energy was sufficiently high for the subsequent biocatalytic reactions at the cathode. The conductive CNT film cathode enabled the efficient sequential transfers of electrons to the Rh-containing mediator and NAD^+^, rapidly regenerating NADH that participated in the enzymatic reaction of GDH. Through the coupled tandem PEC biocatalysis by GDH, l-glutamate was synthesized from α-ketoglutarate (50 mM) and NAD^+^ (2 mM) at an initial conversion rate of 2.4 mM h^−1^ with a high faradaic efficiency of 96.2%. Furthermore, we achieved l-glutamate production yield of 81.5% in a long-term operation for 48 h (TTN_GDH_: 1.08 × 10^5^, TOF_GDH_: 6.2 × 10^3^ h^−1^). Our study opens up possibilities of practical realization of artificial photosynthesis by demonstrating a large-scale, unbiased biocatalytic PEC system that operates solely by solar energy.

## Methods

### Materials

Formamidinium iodide (FAI), methylammonium bromide (MABr), and TiO_2_ paste (30NR-D) were purchased from the Dyesol Ltd (Australia). All other chemicals including β-nicotinamide adenine dinucleotide hydrate (NAD^+^, ≥96.5%), α-ketoglutaric acid disodium salt dihydrate (≥98.0%), ammonium sulfate (≥99.0%), l-glutamate dehydrogenase from bovine liver (GDH, ammonium sulfate suspension), and formate dehydrogenase from *Candida boidinii* (FDH, lyophilized power) were purchased from the Sigma-Aldrich Corp. (USA) and used without further purification.

### FeOOH/BiVO_4_ photoanode fabrication

To prepare the BiOI layer by electrodeposition, the precursor solution was prepared by dissolving Bi(NO_3_)_3_ 5H_2_O (0.04 M) and KI (0.4 M) in distilled water (50 ml) adjusting the pH to 1.7 through addition of HNO_3_ followed by the addition of *p*-benzoquinone solution (0.2 M) in ethanol (20 ml). The solution was vigorously stirred for 30 min. The electrodeposition was performed under −0.1 V of a cathodic bias for 4 min using an as-prepared solution in the conventional three-electrode configuration with fluorine-doped SnO_2_ (FTO) substrates as a working electrode, Ag/AgCl (3 M) as a reference electrode, and Pt mesh as the counter electrode. Afterward, the BiOI layer on the FTO was washed with distilled water several times and dried under air. To convert deposited BiOI into BiVO_4_, a certain amount of DMSO solution containing vanadyl acetylacetonate (0.2 M) was dropped onto a dried BiOI layer, followed by heating at 450 °C for 2 h with a ramping rate of 1 °C min^−1^. After cooling in air, the electrode was soaked in a NaOH aqueous solution (1 M) with mild stirring for 30 min to dissolve excess V_2_O_5_, followed by washing with distilled water and air drying. To deposit the FeOOH oxygen evolution catalyst onto the BiVO_4_ electrode, photoelectrodeposition was carried out in (NH_4_)_2_ Fe(SO_4_)_2_ 6H_2_O (0.1 M) aqueous solution with gentle stirring. In the three-electrode configuration with BiVO_4_ as a working electrode, Ag/AgCl (3 M) as a reference electrode, and Pt mesh as a counter electrode, an external bias of 0.25 V was applied for 10 min with illumination on the glass side of the BiVO_4_ electrode via a 450 W Xe lamp. After the photodeposition, an external bias of 1.2 V was applied under dark for 1 min. The prepared FeOOH/BiVO_4_ electrode was washed with distilled water several times and dried under air.

### Solar cell device fabrication

The device structure consisted of fluorine-doped SnO_2_ (FTO) substrate/bi-layers of TiO_2_ (compact and mesoporous)/perovskite/2,2′,7,7′-tetrakis(*N*,*N*′-di-*p*-methoxyphenylamine)-9,90-spirobifluorene (spiro-OMeTAD)/Au electrode. Fresh FTO substrates were prepared by successive rinsing with acetone, ethanol, and deionized water, followed by UV-ozone treatment for 10 min. The compact TiO_2_ layer was prepared by spin-coating a solution of titanium isopropoxide diluted in ethanol for 30 s at 3000 rpm and subsequent sintering at 500 °C for 30 min. The mesoporous TiO_2_ layer was prepared by spin-coating a solution of Dyesol paste (30NR-D) diluted in ethanol at 4500 rpm for 30 s, followed by annealing at 500 °C for 30 min. The mesoporous TiO_2_ layer was doped with Li^40^, which was achieved by spin-coating of 0.1 M of bis(trifluoromethylsulfonyl)imide lithium salt (Li-TFSI) in acetonitrile at 3000 rpm for 10 s, followed by sintering at 450 °C for 30 min. Two-step spin-coating procedure (initially at 1000 rpm for 10 s and then at 4000 rpm for 30 s) with dripping of anti-solvent (100 µl of chlorobenzene) at 15 s prior to the completion of the spin-coating was used to form the perovskite layers. Post-casting annealing at 100 °C for 10 min was carried out to remove remaining solvent from the film. The perovskite precursor solutions consisted of FAI (1 M), PbI_2_ (1.1 M), MABr (0.2 M), and PbBr_2_ (0.2 M) in anhydrous DMF:DMSO (4:1), and with the CsI stock solution (1.5 M in DMSO) added and stirred for an hour. We used a 0.45-µm PTFE membrane filter to purify the precursor solutions before the spin-coating step. The hole-transporting layer was prepared by spin-coating of spiro-OMeTAD solution (60 mM in chlorobenzene), doped with Li-TFSI and 4-tert-butylpyridine at a concentration of 17.5 and 28.8 µl ml^−1^, respectively, at 3000 rpm for 30 s. Finally, we prepared a gold electrode of 80 nm thickness through thermal evaporation.

### Synthesis of CNT film cathode

A total of 30 ml of ethanol containing 20 mg of MWCNT was homogenized for 20 min and sonicated for 1 h. The MWCNT dispersion was vacuum-filtrated on an anodized aluminum oxide membrane with an average pore size of 100 nm, followed by vacuum drying to fabricate the CNT film. To make the CNT film an electrode, the film was cut into 1 × 1 cm^2^ pieces and was connected by copper wire using silver paste covered with insulating epoxy resin.

### Synthesis of redox mediator, **M**

We synthesized the redox mediator, [Cp*Rh(bpy)H_2_O]^2+^ (Cp* = C_5_Me_5_, bpy = 2,2′-bipyridine) by mixing the Rh-containing precursor with the bipyridine ligand^41^. Briefly, pentamethylcyclopentadienylrhodium (PCD) chloride dimer (97; Sigma-Aldrich) was dissolved in 8 ml of methanol (47 mM), and 2,2′-bipyridine (>99%, Sigma-Aldrich) was dissolved in 5 ml of methanol (160 mM). After 30 min of sonication, the bipyridine solution was added to the PCD solution dropwise with moderate magnetic stirring. The mixed solution became transparent yellow in a few minutes and we kept stirring the solution for 1 h . Then, the mixed solution was added to the 65 ml of diethyl ether and stored in refrigerator over 24 h. The solution was filtered and washed using diethyl ether several times. The collected precipitate was vacuum-dried at room temperature at least for 16 h.

### Photoelectrochemical analysis and regeneration of cofactor

The voltammetric analyses of each electrode were carried out with a potentiostat/galvanostat (WMPG 1000, Wonatech Co., Korea). To examine the water oxidation ability of the FeOOH/BiVO_4_ photoanode, linear sweep voltammetry was conducted in the three-electrode configuration with the FeOOH/BiVO_4_ photoanode as a working electrode, Ag/AgCl (3 M) as a reference electrode, and Pt mesh as a counter electrode in phosphate buffer (0.1 M, pH 7.5) with or without Na_2_SO_3_ as a hole scavenger at a scan rate of 20 mV s^−1^. A 450 W Xe lamp with a 420 nm cut-off filter and an infrared radiation filter was used as the light source, and it was calibrated at 100 mW cm^−2^ on the surface of photoanode using an ILT 1400-A radiometer (International Light Technologies). The tandem configuration was constructed with the glass side of the perovskite solar cell being placed behind the photoanode at a distance of ~1 cm out of reaction chamber to avoid direct contact with the aqueous electrolyte. The exposed FTO of the photoanode and gold electrode on the hole-transporting material of the perovskite solar cell was connected with a copper wire to form ohmic contact. The active areas of the photoanode and perovskite solar cell were defined with metal shadow mask of 1 cm^2^. The electrochemical reduction of the **M** by the CNT film was performed in the three-electrode configuration with the CNT film as a working electrode in the phosphate buffer (0.1 M, pH 7.5) varying the concentration of the **M** and NAD^+^. The photoelectrochemical NADH regeneration was conducted in the two-compartment system connected with a salt bridge as an ion conductor without an applied bias (0 V). The electrolyte of both compartments was phosphate buffer (0.1 M, pH 7.5), and in the cathodic part, **M** (0.5 mM) and NAD^+^ (2 mM) were added.

### Photoelectrochemical biocatalysis

Photoelectrochemical biocatalysis was coupled with the photoelectrochemical regeneration of NADH using the FeOOH/BiVO_4_/perovskite solar cell tandem configuration with the two-electrode configuration. The reaction medium for the enzymatic reaction consisted of NAD^+^ (2 mM), **M** (0.5 mM), α-ketoglutaric acid disodium salt dihydrate (50 mM), (NH_4_)_2_SO_4_ (250 mM), and GDH (40 U) in 3 ml of phosphate buffer (0.1 M, pH 7.5). The l-glutamate production was analyzed by using a 1260 Infinity liquid chromatography system (Agilent Technologies, U.S.A.). TTN_GDH_, TOF_GDH_ were calculated according to Eqs. (2) and (3), respectively:

2 $${\mathrm{TTN}}_{{\mathrm{GDH}}} = \frac{{{\mathrm{Maximum}}\,{\mathrm{concentration}}\,{\mathrm{of}}\,{ {{{{\scriptstyle{\mathrm{L}-}}\mathrm{glutamate}}}}}}}{{{\mathrm{Concentration}}\,{\mathrm{of}}\,{\mathrm{GDH}}}},$$

3 $${\mathrm{TOF}}_{{\mathrm{GDH}}}\,\left({\mathrm{h}}^{- 1} \right){\mathrm{ = }}\frac{{{\mathrm{Concentration}}\,{\mathrm{of}}\,{{{{\scriptstyle{\mathrm{L}-}} {\mathrm{glutamate}}}}} \, {\mathrm{after}}\,{\mathrm{reaction}}\,{\mathrm{for}}\,{\mathrm{the}}\,{\mathrm{first}}\,{\mathrm{hour}}}}{{{\mathrm{Concentration}}\,{\mathrm{of}}\,{\mathrm{GDH}} \times {\mathrm{time}}}}.$$

### Material characterization

The morphology of electrodes was observed using an S-4800 field emission scanning microscope (SEM; Hitachi Co., Japan) and a JEM-3010 transmission electron microscope (TEM; JOEL Ltd, Japan). The crystal structure of the BiVO_4_ electrode was examined using a thin film X-ray diffractometer (XRD; RIGAKU Co. Japan) with a Cu Kα radiation wavelength of 1.5418 Å. UV–vis absorbance and transmittance spectra was obtained using a V-650 UV/visible spectrophotometer (JASCO Inc., Japan). X-ray photoelectron spectroscopic analysis was carried out using a K-alpha (Thermo Scientific, USA) in the scan range of 0–1200 eV. The photoconversion efficiency of the perovskite photovoltaic device was measured using a K3000 solar simulator (McScience, Korea) under solar-simulated AM 1.5G illumination, and it was calibrated using a NREL-certified silicon reference cell.

## Electronic supplementary material

Supplementary Information

## Data Availability

All relevant data are available from the authors upon reasonable request.
