# Supplementary material for: Unbiased biocatalytic solar-to-chemical conversion by FeOOH/BiVO4/perovskite tandem structure
Source: Nat Commun. 2018 Oct 11;9:4208. doi: 10.1038/s41467-018-06687-z (PMC6181951; doi:10.1038/s41467-018-06687-z)
Supplement: Supplementary file 1 — Supplementary Information [file 41467_2018_6687_MOESM1_ESM.pdf]

Supplementary Information

Unbiased Biocatalytic Solar-to-Chemical Conversion by FeOOH/BiVO$_4$/Perovskite Tandem Structure

Lee et al.

Supplementary Figures

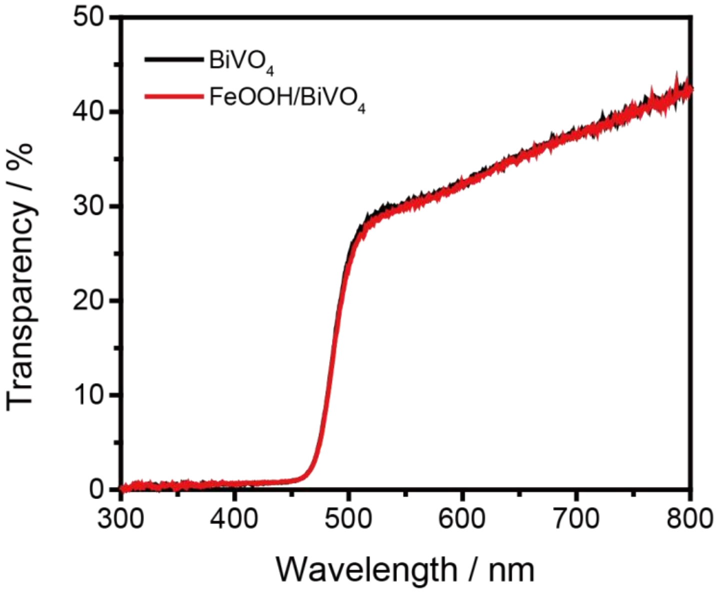

Supplementary Figure 1. Effect of FeOOH deposition on $BiVO_4$ film's optical property. Transparency of bare $BiVO_4$ film (black) and FeOOH-deposited $BiVO_4$ film (red) on the FTO substrate. The deposition of FeOOH catalyst did not affect the transparency of the $BiVO_4$ photoanode.

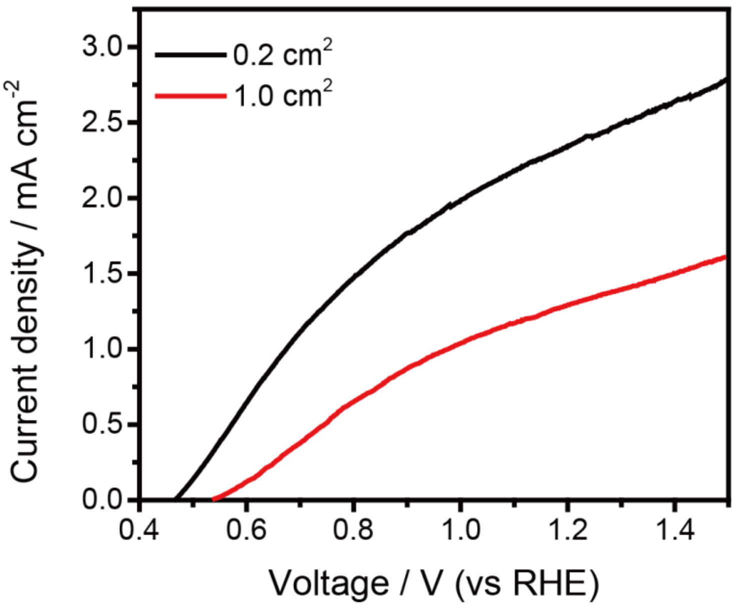

Supplementary Figure 2. Effect of active area on the PEC performance of $BiVO_4$ photoanode. Linear sweep voltammetry of the FeOOH/$BiVO_4$ photoanode under illumination with a shadow mask of 0.2 cm^2 (black) and 1.0 cm^2 (red) on the electrode.

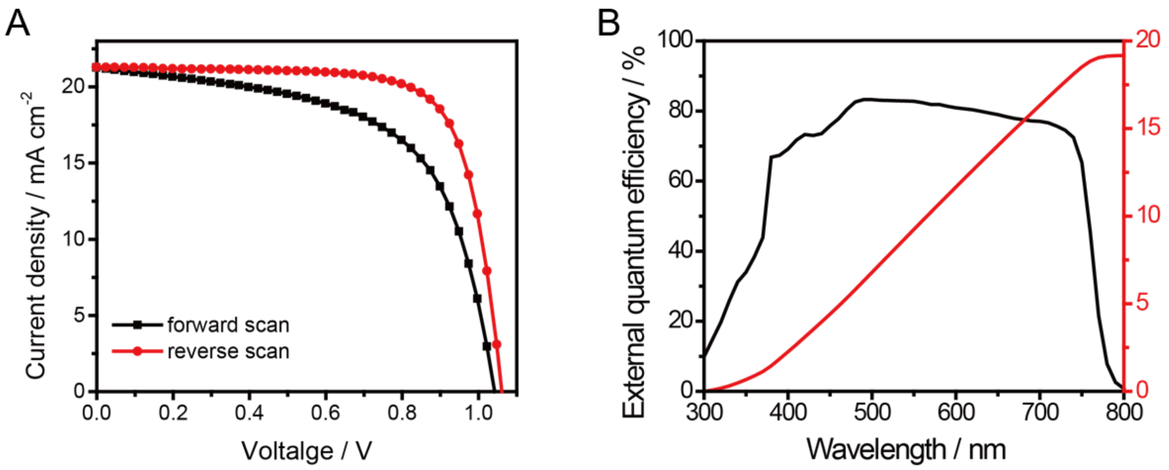

Supplementary Figure 3. Photovoltaic performance of perovskite solar cell. (A) I-V curves of the triple cation perovskite solar cell with an active area of 0.1 cm^2. (B) External quantum yield of the triple cation perovskite solar cell and the integrated short-circuit current density.

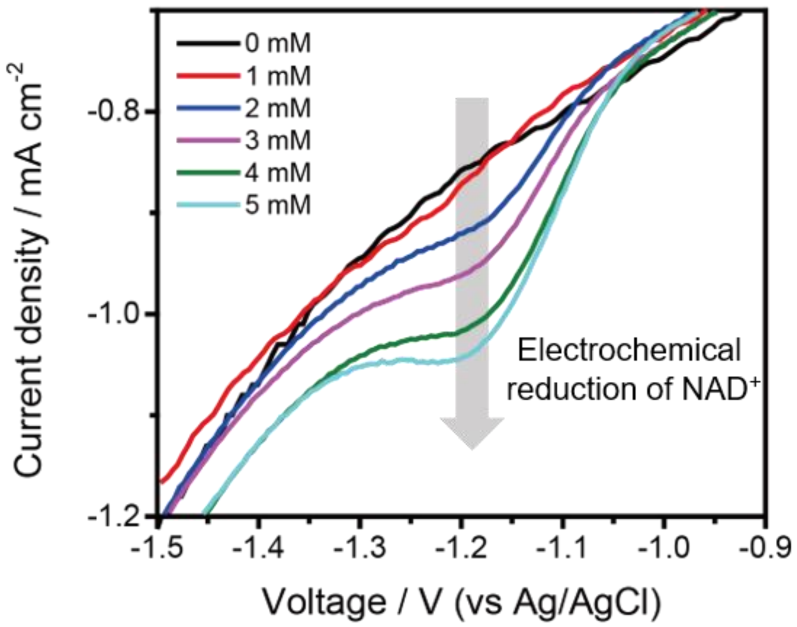

Supplementary Figure 4. Electrochemical reduction of NAD$^+$ on CNT film. Linear sweep voltammetry of CNT film in the phosphate buffer (0.1 M, pH 7.5) containing of NAD$^+$ (0~5 mM) at the scan rate of 50 mV/s.

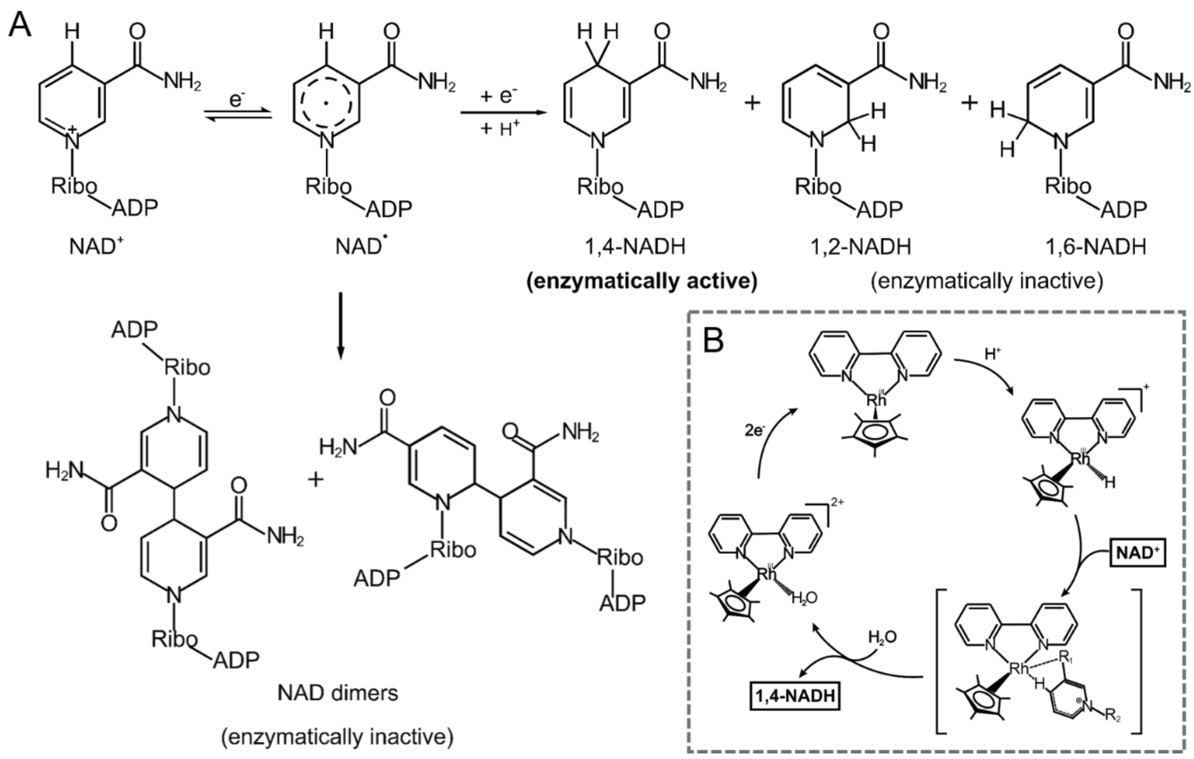

Supplementary Figure 5. Possible pathways for NAD^+ reduction and the regeneration of enzymatically active 1,4-NADH by Rh-based mediator. (A) Schematic representation of the reduction mechanism of NAD^+. Reduced forms of NAD^+ include the enzymatically active 1,4-NADH and enzymatically inactive 1,2-NADH, 1,6-NADH and NAD dimers (NAD_2)[1,2]. (B) Schematic description of the hydride transfer by the Rh complex mediator to NAD^+ producing enzymatically active 1,4-NADH[3].

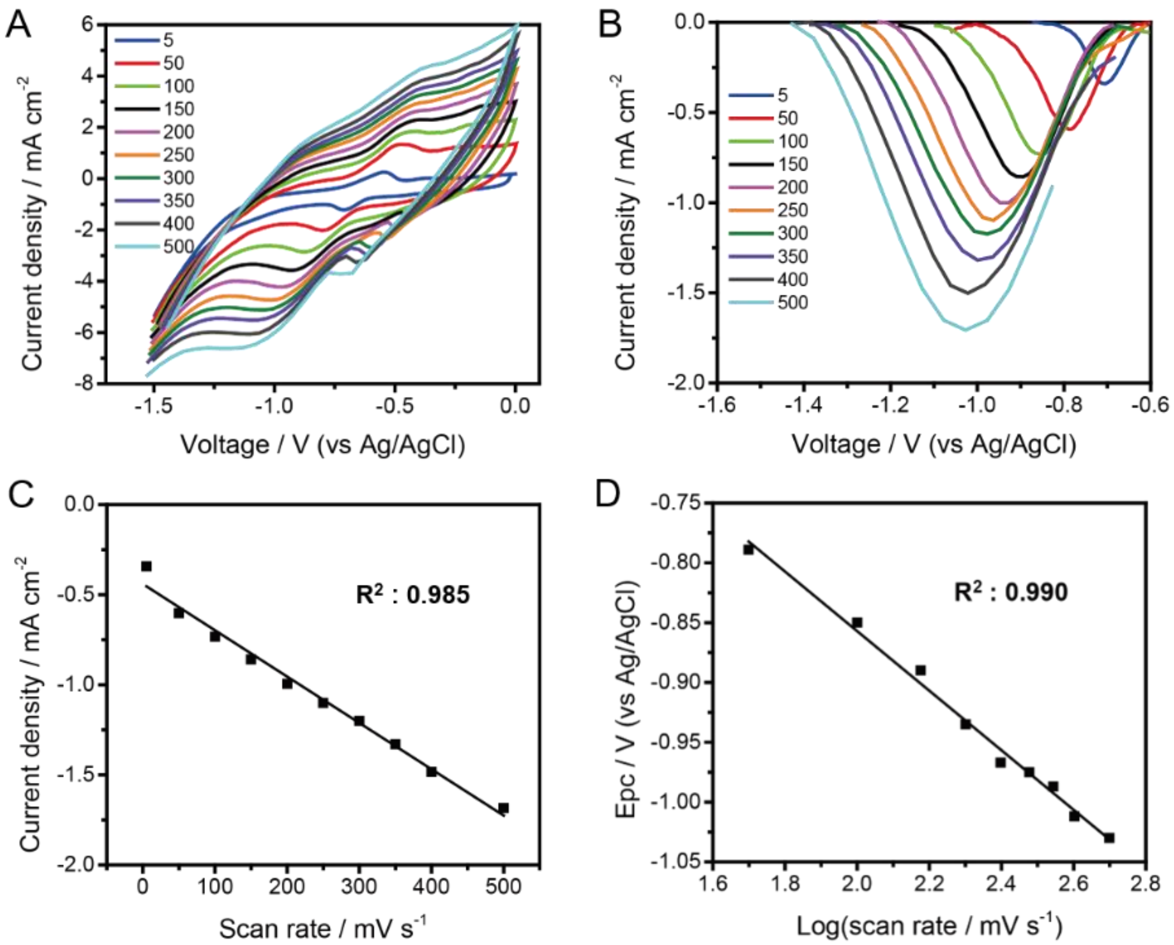

Supplementary Figure 6. Electrochemical analysis of CNT film with **M** at different scan rates (A) Cyclic voltammograms of the CNT film cathode in the phosphate buffer (0.1 M, pH 7.5) containing 0.5 mM of **M** at the range of scan rate from 5 to 500 mV/s. (B) Faradaic current density at the difference scan rate deconvoluted from the cyclic voltammograms in Figure S6A. (C) The relationship between the scan rate and cathodic peak current (I_{pc}). (D) The relationship between the logarithms of the scan rate and the cathodic potential peak (E_{pc})

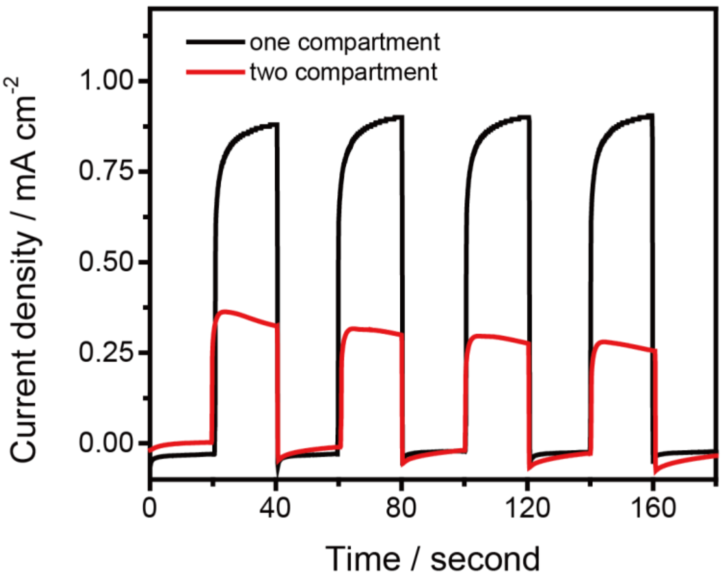

Supplementary Figure 7. Comparison of photocurrent generation by one-compartment tandem system with two-compartment tandom system. Photocurrent profile of FeOOH/$BiVO_4$/perovskite tandem configuration in one compartment (black) and two compartment (red) in a phosphate buffer (0.1 M, pH 7.5) connected by a salt bridge.

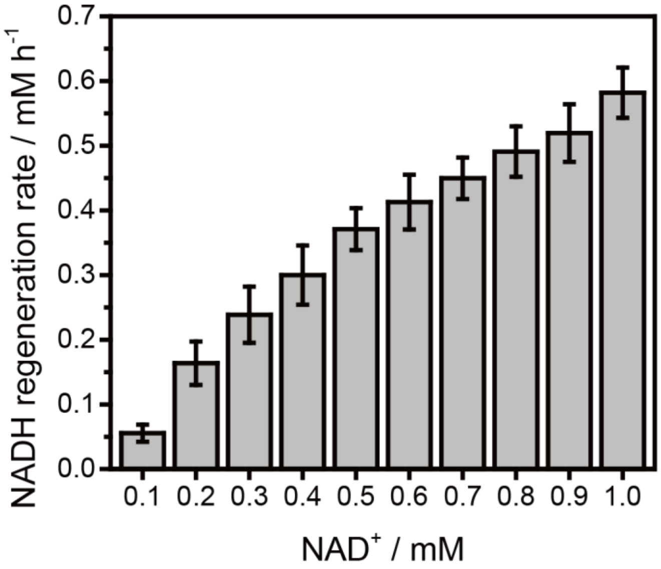

Supplementary Figure 8. Effect of NAD^+ concentration on NADH regeneration rate. The NADH regeneration rate increased with the increasing NAD^+ concentration. Experimental conditions: 0.5 M **M** in a phosphate buffer (0.1 M, pH 7.5) (mean ± standard deviation, n = 3)

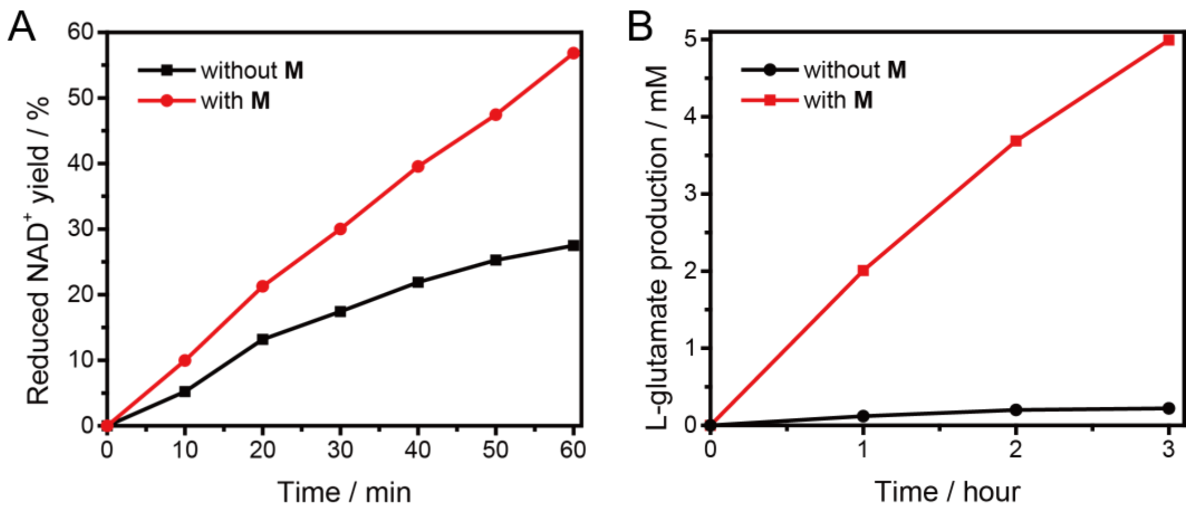

Supplementary Figure 9. Effect of **M** on NADH regeneration and enzymatic reaction. (A) PEC reduction of NAD^+ in the presence and absence of **M**. Reaction conditions: 1 mM NAD^+ in a phosphate buffer (0.1 M, pH 7.5), 0.5 mM **M**. (B) PEC-based GDH-catalyzed production of L-glutamate in the presence and absence of **M**. Reaction conditions: 0.5 mM **M**, 2 mM NAD^+, 50 mM α-ketoglutarate, and 250 mM $(NH_4)_2SO_4$ in a phosphate buffer (0.1 M, pH 7.5).

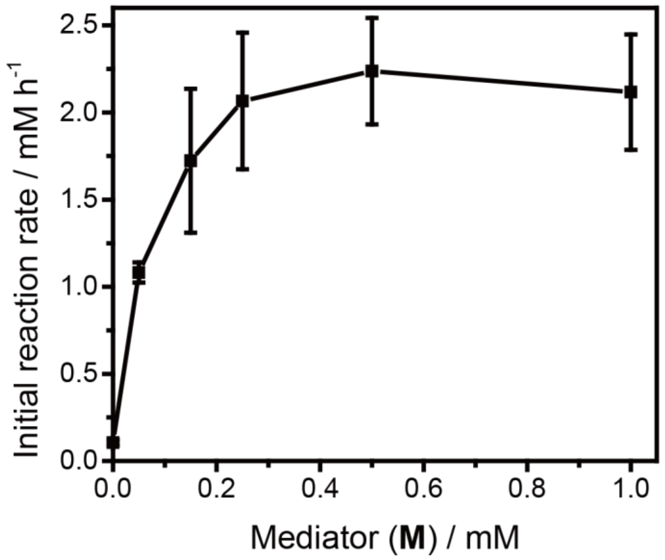

Supplementary Figure 10. Effect of **M** on the performance of biocatalytic PEC system. **M** concentration affects the initial rate of GDH catalysis in the tandem PEC system. Reaction condition: 2 mM NAD^+, 50 mM α-ketoglutarate, and 250 mM $(NH_4)_2SO_4$ in a phosphate buffer (0.1 M, pH 7.5) (mean ± standard deviation, n = 3)

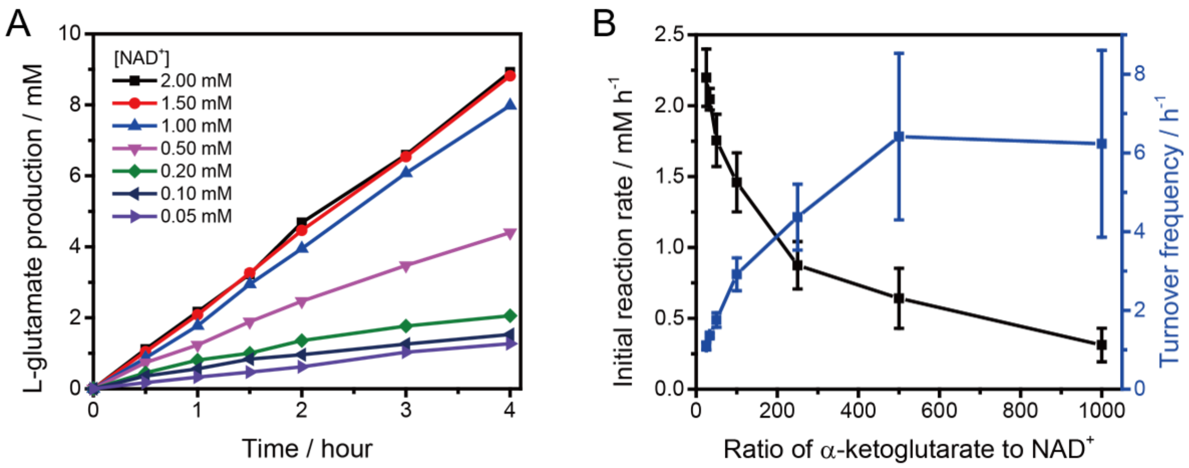

Supplementary Figure 11. Effect of NAD$^+$ concentration on the initial rate of GDH catalysis in the tandem PEC system. (A) GDH-catalyzed production of L-glutamate by the tandem PEC platform with different NAD$^+$ concentration. Reaction conditions: 50 mM α-ketoglutarate, 0.5 mM **M**, and 250 mM $(NH_4)_2SO_4$ in a phosphate buffer (0.1 M, pH 7.5). (B) Effect of α-ketoglutarate to NAD$^+$ ratio on the initial reaction rate and the turnover frequency with respect to NAD$^+$. The turnover frequency of NAD$^+$ = L-glutamate concentration after 1 hour reaction/NAD$^+$ concentration**.** (mean ± standard deviation, n = 3)

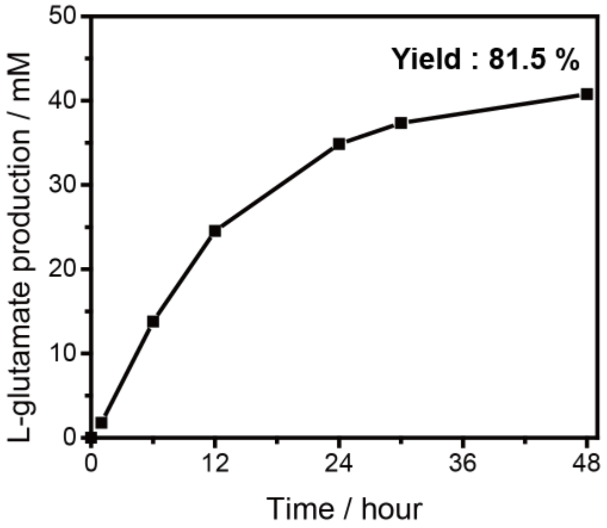

Supplementary Figure 12. Time profile of L-glutamate production by biocatalytic PEC system for two days. Time profiles of GDH-catalyzed production of L-glutamate by the tandem PEC platform. Reaction condition: 2 mM NAD$^+$, 0.5 mM **M**, 50 mM α-ketoglutarate, and 250 mM $(NH_4)_2SO_4$ in a phosphate buffer (0.1 M, pH 7.5).

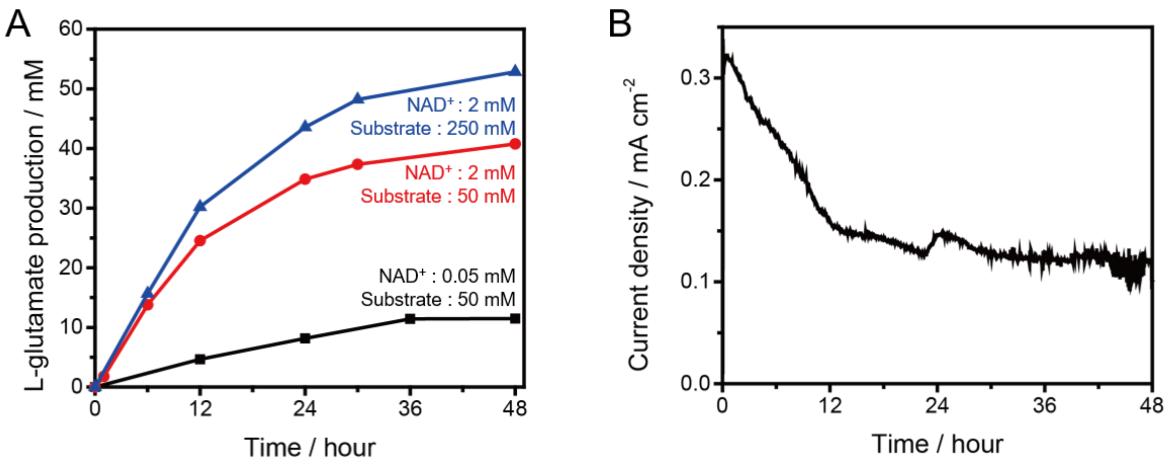

Supplementary Figure 13. Long-term stability of tandem PEC biocatalytic system. (A) Time profiles of GDH-catalyzed production of L-glutamate by the tandem PEC platform with different NAD^+ and substrate concentrations. Reaction condition: 0.5 mM **M**, and 250 mM $(NH_4)_2SO_4$ in a phosphate buffer (0.1 M, pH 7.5) (B) Time profile of the current density of the two-electrode PEC cell during GDH-driven production of L-glutamate. Reaction condition: 0.5 mM **M**, 2 mM NAD^+, 50 mM α-ketoglutarate, and 250 mM $(NH_4)_2SO_4$ in a phosphate buffer (0.1 M, pH 7.5).

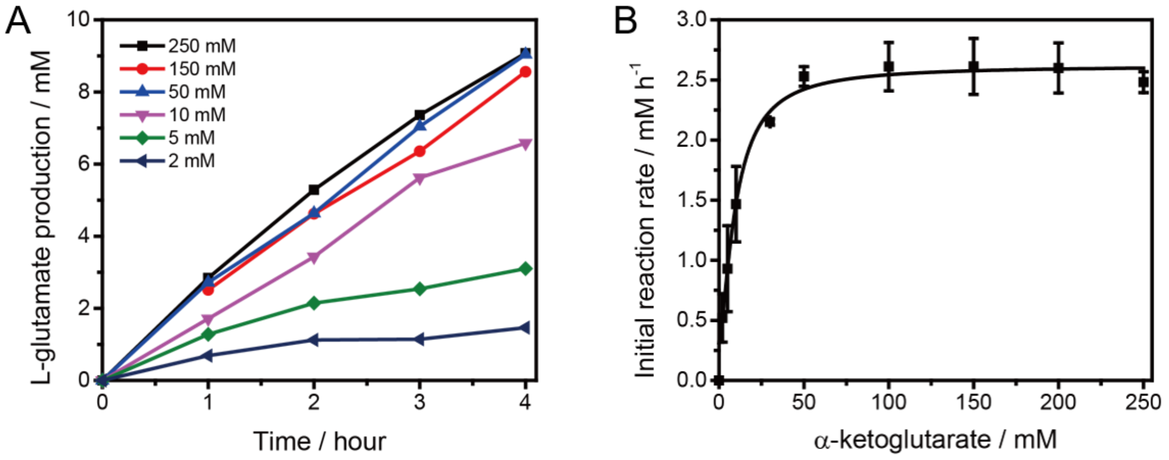

Supplementary Figure 14. Effect of substrate concentration on L-glutamate production by the tandem PEC system. (A) GDH-catalyzed production of L-glutamate by the tandem PEC platform with different substrate concentration. Reaction conditions: 2 mM NAD^+, 0.5 mM **M**, and 250 mM $(NH_4)_2SO_4$ in a phosphate buffer (0.1 M, pH 7.5). (B) Effect of substrate concentration on the initial rate of GDH-driven reaction. Reaction conditions: 0.5 mM **M**, 250 mM $(NH_4)_2SO_4$ in a phosphate buffer (0.1 M, pH 7.5), 1 hour. (mean ± standard deviation, n = 3)

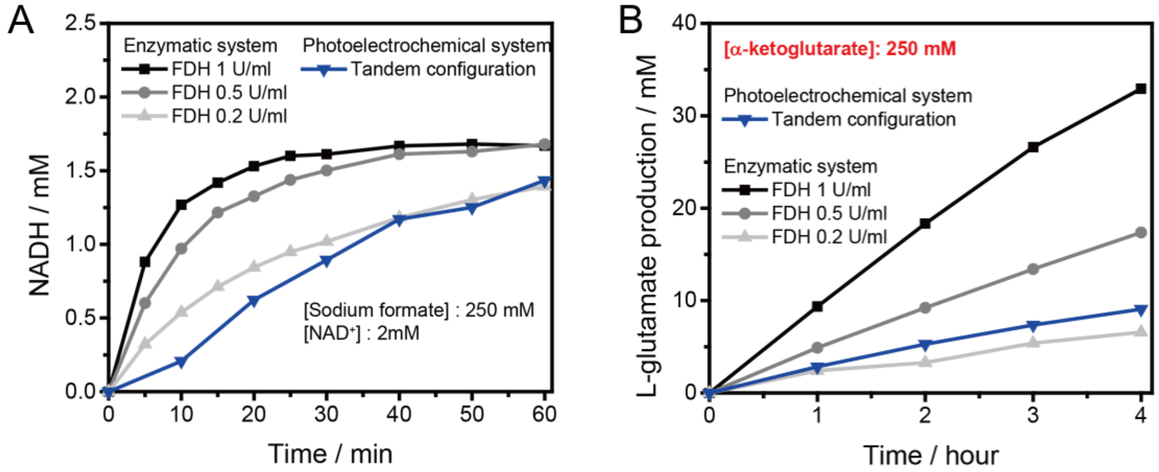

Supplementary Figure 15. Performance comparison between enzymatic cofactor recycling system and PEC-based cofactor regeneration system. (A) NADH regeneration profile by the tandem PEC system compared with those by the enzymatic recycling system using different concentration of formation dehydrogenase. Enzymatic recycling reaction conditions: 2 mM NAD^+, 250 mM sodium formate in a phosphate buffer (0.1 M, pH 7.5). PEC reaction conditions: 2 mM NAD^+, 0.5 mM **M** in a phosphate buffer (0.1 M, pH 7.5). (B) Time profiles of GDH-catalyzed production of L-glutamate by the tandem PEC system and the enzymatic recycling system. Enzymatic recycling reaction conditions: 2 mM NAD^+, 250 mM sodium formate, 250 mM α-ketoglutarate, 250 mM $(NH_4)_2SO_4$ in a phosphate buffer (0.1 M, pH 7.5). PEC reaction conditions: 2 mM NAD^+, 0.5 mM **M**, 250 mM α-ketoglutarate, 250 mM $(NH_4)_2SO_4$ in a phosphate buffer (0.1 M, pH 7.5).

Supplementary References

1. Wang X, Saba T, Yiu HHP, Howe RF, Anderson JA, Shi J. Cofactor NAD(P)H Regeneration Inspired by Heterogeneous Pathways. *Chem* **2**, 621-654 (2017).

2. Bresnahan WT, Elving PJ. The role of adsorption in the initial one-electron electrochemical reduction of nicotinamide adenine dinucleotide (NAD^+). *Journal of the American Chemical Society* **103**, 2379-2386 (1981).

3. Lo HC, Buriez O, Kerr JB, Fish RH. Regioselective Reduction of NAD^+ Models with $[Cp*Rh(bpy)H]^+$: Structure-Activity Relationships and Mechanistic Aspects in the Formation of the 1,4-NADH Derivatives. *Angewandte Chemie International Edition* **38**, 1429-1432 (1999).
